# Decentralized management of laboratory automation

**DOI:** 10.1155/S1463924694000143

**Published:** 1994

**Authors:** Don Chambers

**Affiliations:** Sobering-Plough Research Institute 2000 Galloping Hill Road Kenilworth New Jersey 07033 USA

## Abstract

It is generally believed that successful robot users have dedicated centralized robotic groups. While such a generalization holds some merit historically, the availability of newer more user-friendly robots and workstations in recent years and a more computer-literate work force today is changing the way that automation can be managed. Decentralization recognizes robots and workstations as additional tools for all analysts, not a select few. Such an approach initiates involvement and education of more staff with respect to automation. This further ushers the development of automated methods instead of the automation of manually-developed methods. Decentralization also provides local control of resources to address the priorities of a specific functional group within the department. Both a vision of the future, as well as a look at the past, should be considered when determining how to manage robotic and other means of automation. This paper discusses decentralized management of robots as currently applied and envisioned in a large pharmaceutical analytical R & D department.


Journal of Automatic Chemistry, Vol. 16, No. 4 (July-August 1994), pp. 135-137

### Decentralized management of laboratory automation

**Don Chambers**

*Schering-Plough Research Institute, 2000 Galloping Hill Road, Kenilworth, New Jersey 07033, USA*

*It is generally believed that successful robot users have dedicated centralized robotic groups. While such a generalization holds some merit historically, the availability of newer more user-friendly robots and workstations in recent years and a more computer-literate work force today is changing the way that automation can be managed. Decentralization recognizes robots and workstations as additional tools for all analysts, not a select few. Such an approach initiates involvement and education of more staff with respect to automation. This further ushers the development of automated methods instead of the automation of manually-developed methods. Decentralization also provides local control of resources to address the priorities of a specific functional group within the department. Both a vision of the future, as well as a look at the past, should be considered when determining how to manage robotic and other means of automation. This paper discusses decentralized management of robots as currently applied and envisioned in a large pharmaceutical analytical R & D department.*

### Introduction

A pharmaceutical analytical R & D department is responsible for the development and application of test procedures in order to evaluate the stability and quality specifications for the drug product and its components. A pharmaceutical analytical R & D department will usually function as a 'QC laboratory' for supplies used during preclinical and clinical investigations. This development stage of the novel drug substance occurs during a six to eight year period for any one project. These activities culminate in the submission of a regulatory package of information that supports the commercialization of the product. The environment is dynamic in that project priorities and projects themselves can change on a fairly frequent basis. Only about one in 12 compounds that are recommended for development ever become successful commercial products. Nine of the 12 will never become products because they are clinical failures or are economically unpromising. Two of the 12 will be market failures, and only one of the 12 will be a commercial success [1]. It is rare to have projects in a pharmaceutical analytical R & D department that have large routine sample loads on a daily or even a weekly basis for any one project. Certainly nothing as great as what a QC laboratory could experience with 100 to 200 or more batches per year for a single product. However, large numbers of commercial batches only occur for one in three projects that are envisioned as being a market success. Thus, justifying an R & D investment in robotics based on a potential future return in QC only has a one in three chance of coming to fruition. In the Physical and Analytical Chemisty R & D department at Schering-Plough, it is the summation of clinical supply testing and stability samples that can provide a potential work load for which the investment in automation can be justified. However, the investment must be controlled, and the centralization of the automation effort may not be the best way to support the dynamic nature of the task in a department with diverse automation needs. Furthermore, it should be remembered that gains in productivity are not the only advantages to automation. Improvements in quality of data as well as employee job satisfaction can also be realized [2, 3].

During a presentation at ISLAR 91, Tomlinson [3] said: 'Also noteworthy from our compilation of information was that the successful robot users eventually evolved dedicated, centralized robotics groups that develop the systems to be used as turnkey instruments by staff that are inexperienced in automation'. Such a conclusion appears to be appropriate for work functions that have large routine sample loads. However, the process of automating the analytical laboratory has not yet been completed. Therefore, the optimum way in which to manage robotics may still be evolving, and the concept that centralization is best may only be true for this particular time in the evolutionary cycle or at least only true for some types of laboratories. Interestingly, during ISLAR 84 it was envisioned that: 'Automation specialists are emerging as a new function in the laboratory. This evolution is similar to that which created the need for chemical engineers and/more recently, computer specialists. In the future; as this technology becomes widely used, these specialists will disperse back into the operating organization' [4].

During ISLAR 85 Francis Zenie [5] also noted that: 'The laboratory of the future will be open with islands devoted to integrated systems. Laboratories will be more decentralized with clearer responsibility for final results including precision, cost and turn around time'.

So, where are we in the evolutionary process of laboratory automation, especially with respect to implementation of robotics?

### History

The Physical and Analytical Chemistry Department within Schering-Plough Research Institute has been involved with robotics since 1984. The first Zymark robot was purchased without a specific project in mind. Since that time, it has been noted that under such circumstances the potential for success is limited [6, 7]. However, the

This paper was presented at the 1993 ISLAR, organized by the Zymark Corporation.

© Zymark Corporation 1994

concept was to evaluate the feasibility of using robots in the laboratories. Also, within any organization, one would expect that the R & D staff should be evaluating new technologies and will need to invest time in learning. The individual responsible for the laboratory computer system was given the additional responsibility for robotics. Time had to be split between the two responsibilities and only about 40% of the individual's time was spent on robotics. Several small projects were completed but without a large payoff. This was a result of the changing environment in R & D that can make what was needed yesterday no longer important tomorrow, as well as the large investment of time (three to six months) that was needed to develop useful robotic systems in the 1980s. The major benefit gained from those early days of involvement with robotics was the knowledge acquired by the staff.

Within the Analytical R & D department there are several functional groups such as Dissolution Analysis, Dosage Formulation Analysis, Inhalation Product Analysis and Computer Systems Support. The beginning of decentralized automation management occurred during the late 1980s when the Dissolution Analysis section became the first group outside of the computer systems support area to be involved with their own robot applications even though there was still an aspect of central control. By 1989, two staff members in the Dissolution Analysis area were involved with dissolution robotics and were directed by the manager of that area. The manager of the Laboratory Automation Group was directing the activity of one staff member for development of a robot for testing aerosol as well as a staff member of the Dosage Formulation Analysis areas. Such an arrangement led to success, some disappointment and a new look at how the Institute manages robotics.

The development of the custom aerosol robot required about two years at a total resource (labour and capital expenditures) cost of $700 000. Since 1989, this system and an additional clone of this system initiated in 1993 have resulted in a cost savings in our QC laboratories of about $350 000 a year per robot. Thus, we have certainly had success with our investment in robotics; however, in 1990 it was recognized that it was time to re-evaluate how the automation resources were managed. Those involved with computer support could no longer afford to spend time on robotics. Dilution of one of these individual's efforts had probably doubled the elapsed time required for implementation of the aerosol robot. Direction of staff in other groups by the Computer System Support manager to develop robotic systems was also being met with resistance by the project manager. So it was time for a complete change.

### Decentralization

Computer System Support, Dissolution and Dosage Formulation Analysis became independently responsible for managing their resources for automation. The Dosage Formulation Analysis group began its independent robotic automation efforts by evaluating the advantages of PyTechnology. It was recognized that the Zymate robots of the 80s would not meet current needs, but that a System V robot with PyTechnology and in house programming would provide the type of robot systems that could be quickly adapted to the Institute's changing requirements. PyTechnology provided many preprogrammed sections, and additional PySection software routines have been self-programmed and are now available for use with similar projects. Today, the potential application of robotics to every new project is evaluated. Since the decision to automate rests with the same associate director who is involved with the broad R & D scope of the project's management, information pertaining to the potential success and other details of the project are directly available. There is no need for meetings between a centralized automation group and the end user of the automation. Thus, it is possible to react to project changes and priorities in a much more efficient manner. Given a new assignment, a fully documented and validated robot system for assay sample preparation can be implemented within three weeks.

Computer System Support was divorced from robotics and was focused on the maintenance and further development of the Institute's LAS and LIM systems. This focus has enabled them to expand their services throughout the department and to support the development of a new and expanded LAS/LIM system.

The dissolution group, with knowledge gained from previous experience, was able to more accurately assess their own needs for automation. They have determined that it is more cost effective to use in-house developed automated sampler/analysis systems for extended release dosage forms for which the dissolution runs last from 12 to 24 hours on a set of samples. However, the advantages of a robotic dissolution system are observed when there is the need to test numerous immediate tablet dosage forms. The staff in the dissolution group can self assess which automation technique to apply for options available within their own control, as well as evaluate new systems and determine as to whether their purchase is justified. The resource to program and troubleshoot their systems is available within the group, and each year additional staff become more knowledgeable as to the workings of each available system.

During the past year, Inhalation Product Testing has decided to implement the use of robots to aid in analysing numerous samples that will be generated by projects that are responding to the elimination of fluorocarbon propellants. These robots required some modification of the previously developed aerosol robots, and the new custom systems have been developed in conjunction with Zymark. Again, decentralization has allowed them to directly buy-in to selecting the form of automation and guiding its development so that they can meet the particular project goals.

How robots are managed is as much a function of the culture of the organization as it is specific to management of automation or, more specifically, robotics. Decentralization provides ownership; more members of the analytical staff are directly involved and not just 'looking through the window' at the automation efforts. They now have the ultimate responsibility for the successful application of the automation resources they have chosen. The willingness of employees to make it work is enhanced.

Decentralization encourages participation by all members of the staff. There is no perceived class system in which only an élite few are involved with robotic development. Decentralization can therefore improve employee morale. Staff members see participation in automation as a career development opportunity that can also reduce the routine nature of some of their work.

Communication concerning the project being automated is also improved. Development of the robotic system is within direct control of the department manager responsible for the project. The analyst developing the analytical procedures for the project is also involved directly with the robotic system development simultaneously. The robotic system is more likely to meet the end users needs than one centrally developed by others.

Decentralization provides a broad base of expertise in automation. The loss of one or even two key individuals will not cripple existing or future projects.

### Decentralization: the future

Schering-Plough has now assembled a critical mass of equipment and have trained a sufficient number of our analytical staff to not only run the robots but to program and execute the necessary system validations.

To be truly successful in fully automating the Institute's laboratories, especially in Analytical R & D, the automation techniques must be in the hands of almost everyone, not just a select few. Analytical methods should be developed with automation instead of automation of manually developed methods. How much time and money is now spent in showing equivalence of the robot method to the initially developed manual method? Why not validate the robot system first? Such an approach may be met with some regulatory resistance, since the the FDA has to validate the procedures. However, this is part of the challenge: whenever one is promoting change in how one operates, one must recognize there will be resistance.

Automation is going to reduce the amount of routine work in laboratories. Therefore, the need for employees who are only capable of performing routine tasks will diminish. Halloran and Rulon have noted that those involved in developing robotic applications need to have skills associated with programming and engineering as well as chemistry [7, 8]. The talents and abilities of almost all laboratory employees will need to reflect these needs. Chemists graduating from college in the 90s are certainly more computer literate that those of the 70s. Training can also be an answer to providing staff members with the proper tools. Some may look at training as a unique additional cost of automation. Training is an additional cost, but it is a cost associated with the application of any useful technology. HPLC was introduced into most pharmaceutical laboratories about 20 years ago and yet today many of us still spend money on HPLC courses for some of our employees each year. These costs never end.

Vendors of laboratory automation will also need to continue to strive to develop products that reduce the learning curve and its time course. Simpler programming languages, efficiency in establishing new programs and the development of focused yet useful workstations are essential to reducing the barriers to decentralization.

### Conclusion

There is no single best way to manage laboratory automation. A manager's own knowledge, the experience of others, the nature of his/her projects and an understanding of her/his own corporate culture are major factors which will influence the way in which he/she determines to manage the automation function. Technology and people are both constantly changing and require that we periodically reconsider how we manage our resources for optimum performance. Managers should consider their own alternatives and make their own decisions and not merely follow the successful path of others whose projects, culture, and staff may be significantly different.

### Acknowledgements

Larry Lorenz, Bob Foester and Dr Ed Mularz are thanked for their helpful discussions.

### References

1. Eastman, H. P., *IMPACT—The Economic and Social Impact of New Jersey's Health Products Manufacturing Industry: New Jersey's Economic Leader* (The New Jersey Health Products Council, Union, New Jersey, 1992).
2. Brunner, L. A., in *Proceedings of the International Symposium on Laboratory Automation and Robotics 1991* (Zymark Corporation, Hopkinton, 1991).
3. Tomlinson, J. J., in *Proceedings of the International Symposium on Laboratory Automation and Robotics 1991* (Zymark Corporation, Hopkinton, 1984).
4. Zenie, F. H., in *Proceedings of the International Symposium on Laboratory Automation and Robotics 1984* (Zymark Corporation, Hopkinton, 1984).
5. Zenie, F. H., in *Proceedings of the International Symposium on Laboratory Automation and Robotics 1985* (Zymark Corporation, Hopkinton, 1985).
6. Scheffler, R., in *Proceedings of the International Symposium on Laboratory Automation and Robotics 1991* (Zymark Corporation, Hopkinton, 1985).
7. Halloran, K. J., in *Proceedings of the International Symposium on Laboratory Automation and Robotics 1992* (Zymark Corporation, Hopkinton, 1992).
8. Rulon, P. W., in *Proceedings of the International Symposium on Laboratory Automation and Robotics 1991* (Zymark Corporation, Hopkinton, 1992).

